# Inaugural bone metastases in non-small cell lung cancer: a specific prognostic entity?

**DOI:** 10.1186/1471-2407-14-416

**Published:** 2014-06-10

**Authors:** Mélanie Deberne, Stanislas Ropert, Bertrand Billemont, Catherine Daniel, Jeanne Chapron, François Goldwasser

**Affiliations:** 1 Radiation Oncology Department, Institut Curie, 26 rue d’Ulm, Paris 75005, France; 2 Oncology Department, Hôpital Cochin, Paris, France; 3 Oncology Department, Institut Curie, Paris, France; 4 Respiratory Medicine Department, Hopital Cochin, Paris, France; 5 Paris Descartes University, Paris, France

**Keywords:** Bone metastasis, Skeletal-related events, Lung adenocarcinoma, Spinal epidural metastasis

## Abstract

**Background:**

In non-small cell lung cancer patients (NSCLC), median survival from the time patients develop bone metastasis is classically described being inferior to 6 months. We investigated the subcategory of patients having an inaugural skeletal-related-event revealing NSCLC. The purpose of this study was to assess the impact of bone involvement on overall survival and to determine biological and tumoral prognosis factors on OS and PFS. An analysis of the subgroup of solitary bone metastasis patients was also performed.

**Methods:**

In a population of 1208 lung cancer patients, 55 consecutive NSCLC patients revealed by inaugural bone metastasis and treated between 2003 and 2010, were retrospectively analysed. Survival was measured with a Kaplan-Meyer curve. Univariate and multivariate analysis were performed using the Stepwise Cox proportional hazard regression model. A p value of less than 0,05 was considered statistically significant.

**Results:**

Estimated incidence of revealing bone metastasis is 4,5% among newly diagnosed lung cancer patients. Median duration of skeletal symptoms before diagnosis was 3 months and revealing bone site was located on axial skeleton in 70% of the cases. Histology was adenocarcinoma (78%), with small primary tumors Tx-T1-2 accounting for 71% of patients. Rate of second SRE is 37%.

Median overall survival was 8.15 months, IQR [5–16 months], mean survival 13.4 months, and PFS was 3.5 months. In multivariate analysis, variables significantly associated with shortened survival were advanced T stage (HR = 2.8; p = 0.004), weight loss > 10% (HR = 3.1; p = 0.02), inaugural spinal epidural metastasis (HR 2.5; p = 0.0036), elevated C-reactive protein (HR = 4.3; p = 0.002) and TTF-1 status (HR = 2.42; p = 0.004). Inaugural spinal epidural metastasis is a very strong adverse pronostic factor in these cases, with a 3 months median survival. Single bone metastasis patients showed prolonged survival of 14.2 months versus 7.6 months, only in univariate analysis (HR = 0.42; p = 0.0059).

**Conclusion:**

Prognosis of lung cancer patients with inaugural SRE remains pejorative. Accurately estimating the survival of this population is helpful for bone surgical decision-making at diagnosis. The trend for a higher proportion of adenocarcinoma in NSCLC patients should result with an increasing number of patients with inaugural SRE at diagnosis.

## Background

Metastatic lung cancer accounts for approximately 58% of newly diagnosed lung cancer as described by a large french prospective epidemiological study conducted in 2010 [1].

It has been estimated that 30% to 65% of patients with metastatic lung cancer will develop bone metastases [2] and median survival from the time patients develop bone metastasis is classically considered as less than 6 months [3].

However, with the introduction of new therapeutic agents such as antiangiogenic therapies or EGFR inhibitors, especially in adenocarcinoma, median survival for patients with advanced stages has increased from approximately 6 months to 12 months [4] thereby extending their disease course and potentially increasing the risk of subsequent skeletal-related-events (SREs). SREs are defined as pathologic fractures, spinal cord compression, a requirement for radiation or surgery to the bone, and malignant hypercalcemia, leading to significant morbidities or are associated with shortened survival.

Few data are available regarding the prognosis of patients having an inaugural SRE in non small cell lung cancer (NSCLC). Sugiura et al. reported a median survival of 7.2 months from the time patients develop bone metastasis in the disease course [5] and a recent study by Bae et al., assessing pronostic factors for 196 non-small cell lung cancer with bone metastasis at the time of diagnosis, showed that ECOG performans status 0–1 and single metastasis were associated with prolonged survival for these patients with synchronous bone metastasis [6].

Lung cancer patients presenting a SRE at diagnosis are challenging, and require a multidisciplinary therapeutic approach, both systemic and local, on the bone disease. Accurately estimating the survival of these population is also helpful for surgical decision-making [7,8].

In this study, we retrospectively analysed 55 NSCLC patients revealed by an inaugural SRE. Our first objective was to investigate the influence of bone metastatic involvement on overall survival. Secondary aims were to report epidemiologic characteristics of this population and to assess clinical and biological prognosis factors of survival and progression-free-survival. We also performed an analysis of patients with single bone metastasis.

## Methods

### Study population

From our department databases, we identified 55 patients with NSCLC revealed by inaugural bone metastasis who were treated between March 2003 and January 2009 at the Oncology Department of Cochin Hospital (Paris) and Institut Curie (Paris). The last follow-up evaluation was performed in January 2012. At the time of final survival analysis, two patients were alive.

Median follow-up is 8.3 months and survival interquartile range (IQR) is [5–16 months].

Variables considered for analysis were: patients demographics, smoking history, weight loss, duration of bone symptoms at diagnosis and OMS status, TNM according to UICC 1997, TTF-1 status, presence and sites of visceral involvement and treatment schedule (chemotherapy, biphosphonates).

Bone disease characteristics were assessed with total number and sites of bone metastasis, location of revealing bone lesion and predominant symptom revealing the lesion (defined as pain, neurologic symptom, hypercalcemia, fracture, or spinal epidural metastasis (SEM), as well as occurrence of a second SRE.

### Ethics approval

This retrospective study has received the approval of the ethic comittee of Paris Descartes University, France, and been carried out in compliance with the guidelines of the Helsinki Declaration of 1975. Clinical informations were anonymized for statistical work-up.

### Statistical analysis

The primary outcome of the study was to investigate the impact of the bone metastatic disease on overall survival and PFS. Survival and disease control from the beginning of the treatment to the date of last follow-up or event were measured with a Kaplan-Meyer curve. Statistical differences between curves were calculated by using the log-rank test for the putative prognostic factors. Comparisons between groups were made using the Pearson or maximum-likelihood test for categorical data and the Student t test for comparison of means. Univariate and multivariate analysis were performed using the stepwise Cox proportional hazard regression model. A p value of less than 0,05 was considered statistically significant.

## Results

### Incidence of bone metastasis revealing lung cancer and patient characteristics

On 349 NSCLC patients treated at Cochin Hospital during this period, 27% (94) patients had synchronous or metachronous bone involvement, and 12% (42) had bone metastasis as first manifestation of their lung cancer. At Institut Curie, the estimated rate of bone metastasis revealing NSCLC is 2% on 859 patients. Baseline characteristics of patients are shown in Table 1. Median age was 62.5 years at diagnosis – with a range of 31 to 92 years. The histologic subtype of NSCLC was adenocarcinoma (78%), squamous cell carcinoma (3.6%) and large cell carcinoma (18.2%).

**Table 1:** Patients characteristics

**Characteristics**	**n = 55**
**Gender (Male/Female)**	65.5%/ 34.5%
***Age (yr, mean)***	62.5
***Histology***	
Adenocarcinoma	78.2%
Squamous cell carcinoma	3.6%
Large cell carcinoma	18.2%
***TTF-1 status***	
+	56%
-	44%
***TNM stage***	
Tx/T1/T2/T3/T4	10.2%/43%/18.4%/20.4%/8.1%
N0-1/ N2/ N3	56.5%/30.4%/13.1%

The mean duration of skeletal symptoms before diagnosis is 5 months. Thirty-six patients- 65.5%- had multiple skeletal lesions and nineteen patients -34.5%- one bone lesion, independently of the visceral metastatic status. Twenty-five patients - 45.5% - had exclusive bone dissemination without visceral disease; among them ten patients -18%- presented a single bone lesion.

Revealing bone site was located on axial skeleton in 70% of cases: vertebra in 34.5%, pelvis in 34.5%, while extra-axial metastasis involved scapula (16.4%), long bone like humerus or femur (16.4%) and ribs (3.6%). Among spine localizations, thoracic level is the first revealing site (63%), followed by lumbar level (33%); only one patient suffered from a C7 lesion accompanied by a C7-D1 cervico-brachial neuralgia. Revealing symptoms were: bone pain (78%), spinal epidural metastasis or cord compression (14.5%) and pathologic fracture (7.2%). No symptomatic malignant hypercalcemia was observed, but biological hypercalcemia was noted in 22% of cases. Therapeutic features are described in Table 2.

**Table 2:** Bone disease management and systemic treatment

**Therapeutic on first SRE**	**n - %**
Radiotherapy	38 - 70.4%
Surgery followed by radiotherapy	9 - 14.8%
Percutaneous vertebroplasty/Medical treatments	2/6 - 14.8%
Biphosphonates	33 - 60%
**Systemic treatment**	
First line chemotherapy	53 - 96%
Second line chemotherapy	31 - 56%

### Analysis of predictive risk factors for overall survival, progression-free-survival and skeletal-related-events

**Figure 1:**
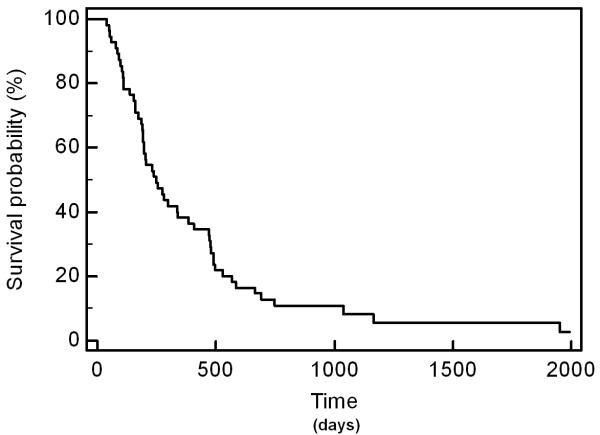
Overall survival according to Kaplan-Meyer.

Median survival obtained by the non parametric method of Kaplan and Meyer was 8.15 months, and mean survival 13.4 months (Figure 1). Survival interquartile range is IQR [5–16 months]. The actuarial 6 months, 1 and 2-year survival rates are respectively 69%, 32% and 9%. Presence of revealing bone site located in spine (HR = 1.72; p = 0.054) and spinal epidural metastasis (HR = 2.4; p = 0.017) were significantly associated with a decreased survival in univariate analysis.

Univariate survival analysis showed as highly significant prognostic factors for longer survival the following pretreatment characteristics (Table 3): Performans status 0–1 (HR = 2.1; p = 0.007), weight loss less than 10% (HR 2.58; p = 0.002), positive TTF-1 status (HR = 0.54; p = 0.03), early stage T0-T1 versus T2-3-4 (HR = 2.37; p = 0.0013), and absence of visceral involvement (HR = 1.8; p = 0.025). The subgroup of patients with no visceral metastasis had a median survival of 12.6 months versus 6.45 months (HR = 1.8; CI 1-3.13; p = 0.025).

**Table 3:** Univariate analysis of clinical and histological parameters on overall survival

**Tumoral and patients characteristics**	**N**	**OS (months)**	***HR***	***p***
OMS 0-1	32	*12*	*2.10*	*0.007*
Vs 2-3	21	*6.2*	*CI 95% 1.11-3.9*	
Weight loss ≥ 10%	12	*3.4*	*2.58*	*0.002*
< 10%	41	*11*	*CI 95% 1.06-6*	
Adenocarcinoma	43	*8.3*	*0.93*	*0.84 - NS*
Vs others histological subtypes	12	*7.8*	*CI 95% 0.49- 1.8*	
TTF1 negative	22	*6.4*	*0.54*	*0.03*
Vs positive	28	*15.4*	*CI 95% 0.29- 1*	
T0-1	26	*15.5*	*2.37*	*0.0013*
vs T2-3-4	23	*6.6*	*CI 95% 1.25-4.4*	
No visceral involvement	25	*12.6*	*1.8*	*0.025*
Vs visceral involvement	30	*6.4*	*CI 95% 1–3.13*	
Solitary bone metastasis	10	*14.2*	*0.42*	*0.0059*
Vs multiple bone and/or visceral metastasis	45	*7.6*	*CI 95% 0.23-0.73*	
Revealing site: spine	19	*6,3*	*1.72*	*0.054*
vs other sites	36	*10*	*CI 95% 0.90-3.27*	
Spinal epidural metastasis				
Yes	9	*3*	*2.4*	*0.017*
No	46	*9,7*	*CI 95% 0.83-5.95*	

The number of bone metastases, independent of visceral involvement status, does not reach significativity for survival in univariate analysis: the group of 19 patients with one bone lesion experienced a median survival of 9.7 months versus 7.6 months for those with multiple skeletal lesions (p =0.11). Therefore, the subgroup of 10 patients having a single bone metastasis without visceral disease has a significantly more favourable median survival of 14.2 months versus 7.6 months for the rest of the population (HR = 0.42; CI95% 0.23-0.73; p = 0.0059).

Pretherapeutic biological parameters associated with increased survival are listed in Table 4.

**Table 4:** Univariate analysis of biological variables on overall survival

**Biological parameters**	***N***	***OS ( months)***	***HR***	***p***
Leucocytes				
≥10 000/mm^3^	23	5.2	2.23	0.0031
<10 000/mm^3^	30	12.6	*CI 95% 1.02-4.15*	
Neutrophils				
< 8000/mm^3^	38	12.6	3.08	0.0001
≥ 8000/mm^3^	15	4	*CI 95% 1.36-7*	
Lymphocytopenia				
Yes	26	7.6	1.53	0.11- NS
No	27	15.6	*CI 95% 0.87-2.7*	
Hemoglobin				
<11 g/dl	9	6.5	0.39	0.0066
≥ 11 g/dl	44	11	*CI 95% 0.14-1.09*	
Albuminemia				
≥ 35 g/L	32	14.5	0.27	0.0001
<35 g/L	10	5.22	*CI 95% 0.09-0.84*	
Alkaline phosphatases				
< 100 UI/L	20	11.2	1.93	0.0165
≥ 100 UI /L	35	6.4	*CI 95% 1.12-3.32*	
Corrected calcemia				
≥ 2,60 mmol/L	11	5	2.38	0.008
< 2,60 mmol/L	38	11.2	*CI 95% 0.96-5.88*	
C-Reactive Protein				< 0.0001
≥ 7 mg/L	35	6.45	4.3	
< 7 mg/L	11	34	*CI 95% 2.38-7.8*	

Finally, in multivariate analysis (Table 5), variables significantly associated with decreased overall survival were T stage (HR = 2.8; p = 0.004), weight loss more than 10% (HR = 3.1; p = 0.02), inaugural spinal epidural metastasis (HR 2.5; p = 0.0036), elevated C-reactive protein (HR = 4.3; p = 0.002) and negative TTF-1 status ( HR = 2.42; p = 0.004).

**Table 5:** Multivariate analysis of clinical and biological parameters on overall survival

	**HR**	**p**
T0-1 vs T 2-3-4	2.8	0.004
Weight loss > 10%	3.1	0.02
TTF-1 status	2.42	0.004
Spinal epidural metastasis	2.5	0.0036
C-Reactive protein	4.3	0.002

We also analysed the prognostic factors associated with systemic progression-free-survival under first line chemotherapy- all types of progression (skeletal or visceral) were recorded. Median PFS for the entire population is 3.5 months.

In univariate analysis, the same variables found to be pronostic for overall survival were discriminant for progression-free-survival such as T stage (HR = 2.16, CI95% 1.16-4; p = 0.0042), TTF-1 status (HR =0.41, CI95% 0.21-0.8; p = 0.04) and performans status (HR =2.06, CI95% 1.1-3.8; p = 0.006), albuminemia ≥35 g/L (HR = 0.41, CI95% 0.15-1.13; p = 0.014), and a C-Reactive Protein under 7 mg/L (HR = 2.66, CI95% 1.47-4.8; p = 0.001).

In multivariate analysis, the only clinical parameter found to be prognostic for PFS is the presence of visceral metastasis (HR = 2.8, CI95% 1.44-5.7; p = 0.0029), leading to a decreased PFS of 4.8 months versus 6.2 months.

### Second skeletal-related event: rate and description

The occurrence rate of second SRE is 37% (20 patients/55), arising in a median delay of 3.7 months.

The repartition of second SRE was: radiation therapy for analgesic purpose (11 pts), spinal chord compression (7) with paraplegia, one fracture, one malignant hypercalcemia. Therapeutic on this second SRE was radiation therapy in 60% cases, surgery for two patients, and medical treatments for the 30% others. Two patients who had been irradiated for a C7 and T7 instable vertebral involvement underwent surgery by laminectomy in a delay of 30 days after radiotherapy, due to neurologic dysfunction.

No characteristic of the initial bone disease could be correlated with the risk of occurrence of second SRE (i.e. axial versus peripheral involvement, number of bone lesions or administration of biphosphonates). Baseline high rate of alcaline phosphatasis was significant for a shorter survival (p = 0,016) in univariate analysis, but was not predictive for the occurrence of a second SRE.

In this limited serie, biphosphonates did not seem to impact the occurrence of second SRE, since 43% of the patients who received biphosphonates had experienced a second SRE versus 31% in the group without biphosphonates, all these data being non-significant. This fact mainly reflects the under-prescription of biphosphonates before 2008.

### Subgroup analysis of patients with single bone metastasis

The subgroup of 10 patients having a single bone metastasis without visceral involvement has a significantly more favourable median survival of 14.2 months versus 7.6 months for the rest of the population (HR = 0.42; CI95% 0.23-0.73; p = 0.0059). This subgroup presents also a prolonged median time to progression of 6.8 months versus 3.5 months ( HR = 0.54, CI95% 0.3-0.96; p = 0.0443). Their frequency of second skeletal event is 58%, probably due to their prolonged survival. Eighty percent of these single bone metastasis are located on pelvis, scapular belt, long bone or thoracic chest, demonstrating that vertebral involvement is more often linked with a polymetastatic disease. On ten patients, eight had a biopsy-proven histology on the bone.

Repartition and treatment of these solitary skeletal metastasis were as follow:

– Metastasis on the humeral glene ( 1 pt), treated by resection, full humeral prosthesis and adjuvant radiotherapy 30 Gy/10 fr.

– Metastasis of the coxo-femoral articulation or femoral bone (2pts), treated by total hip replacement followed by adjuvant radiotherapy 30 Gy/10 fr.

– Metastasis of the radial bone with a pathologic fracture (1 pt), treated by osteosynthesis and adjuvant radiotherapy (30 Gy/10 fr)

– T7 spine metastasis was treated by radiotherapy (1 pt) followed by laminectomy due to spinal chord compression. L3 metastasis in one patient without neurologic impairment was treated by exclusive radiotherapy 30 Gy/10 fr.

– Osteolytic metastasis located on scapula or coracoid apophysa (3 pts) were treated by exclusive radiotherapy (30 Gy/10 fr).

– One patient with a biopsy-proven of a sole bone metastasis of the 4^th^ rib received also exclusive radiotherapy.

In this subgroup, two non-smoker female patients were long survivors at 5 years from the diagnosis. Both of these patients had received a locoregional therapy with lobectomy on the primary lung cancer, and had been treated in a curative intent on their bone metastasis, located on distal extremities (humeral glene and femoral bone). Adjuvant radiotherapy was performed in both case. No relapse occurred in the primary bone site, underlying the fact that a durable bone response can be obtained with a multimodal approach.

## Discussion

Inaugural SRE in NSCLC patients remains of pejorative prognosis, with a median survival of 8.5 months and mean survival of 13.4 months. Our study indicate that locally advanced thoracic T-stage, weight loss > 10%, elevated C-reactive protein at diagnosis, and TTF-1 status are independent prognosis factors for survival.

If absence of visceral spreading, and/or solitary bone metastasis appear to be variables associated with longer survival respectively of 12.6 and 14.2 months in univariate analysis, these trend is not meaningful in multivariate analysis.

To our knowledge, one study have focused on skeletal metastases as the primary symptom revealing lung cancer [9], reporting an incidence of bone metastases revealing lung cancer of 2.4% on a population of 1062 patients treated between 1976 and 2001, while we report an incidence of 4.5%. Adenocarcinoma is the first histologic subtype represented in our serie, accounting for 78% of our patients. Kagohashi in 2003 reported an adenocarcinoma rate of 67% on 24 patients with revealing bone metastasis [9].

Incidence of adenocarcinoma is rising on the lasts epidemiologic reports -accounting for more than 45% of the newly diagnosed cases - and has become the first histology in women, independently of the smoking status [10,11]. It has a tropism for bone dissemination: metastatic spreading in lung cancer differs according to histologic subtype and 45% of patients having adenocarcinoma are metastatic to bone [12]. Interestingly, most of our patients present small primary tumor (Tx-T1-T2 accounts for 71% of patients), with node involvement cN0-N1 showing that bone metastases are more related to early spreading in the bone than to primary tumour volume.

Revealing bone site is mainly located on axial skeleton (70%). Our findings are comparable to previous observations, where 70% of symptomatic lesions are located in thoracic vertebra, 20% in lumbar vertebra and only 10% in the cervical spine [13,14].

Our reported cumulative survival rates at 6 months, 1 and 2 years are very closed to those reported by Sugiura [5] assessing prognostic factors in a serie of 118 patients with bone metastases from lung cancer (59.9% at 6 months, 31.6% at 1 year, and 11.3% at 2 years) and a median survival of 7.2 months.

It has been described how the incidence of SRE in the disease course of NSCLC patients strongly affects survival. In a study of Tsuya et al. [14] on 135 patients with stage IV NSCLC, median survival for the patients experimenting a SRE was 6 months versus 1 year, although not significant, and median survival after SRE was 4.5 months. In the medico-economic study by Delea [15] conducted on 534 patients of whom 55% had experienced a SRE, median survival after the first SRE was 4.1 months.

In surgical series focusing on skeletal metastases in lung cancer, Weiss et al. report a median survival time after surgery of 3 months, and a cumulative 12-months survival after surgery of 13% [7]. Nathan et al. found that in patients with various histologic types of bone metastases, lung cancer patients had the shortest median survival time of 4 months [8]. These surgical series illustrate the major difficulty in selecting NSCLC patients who are likely to benefit from heavy surgical procedures. In attempting to accurately assess the survival expectancy of bone metastatatic patients, orthopaedic surgeons have established a scoring system for the preoperative evaluation of skeletal metastasis [16,17]. Katagiri et al. reported five prognostic factors discriminant for survival, namely, site of primary lesion, performans status, presence of visceral or cerebral metastasis and multiple skeletal metastases [16]. Forsberg has recently developed and validated BETS models (Bayesan Estimated Tools for Survival at 3 months and 12-months) including data such as hemoglobin concentration, preoperative absolute lymphocyte count, ECOG performans status, presence of a complete pathologic fracture, number of bone metastases and primary oncologic diagnosis [17].

Regarding surgical outcomes, we showed that inaugural spinal epidural metastasis (SEM) strongly impact outcome, with a median survival of 3 months for the nine patients who suffered from SEM, as Schiff et al. who reported a median survival of 2.75 months after laminectomy for inaugural SEM [18].

We also confirmed some biological parameters already known to be predictive of pejorative survival, such as hemoglobin rate, neutrophil and white blood cell count [19,20], whereas lymphocytopenia was not statistically significant. Moreover, we found that pretherapeutic C-reactive protein level below 7 mg/L was strongly correlated with survival in multivariate analysis. In a study conducted by Wilop on 210 patients with NSCLC stage IV, evolution of CRP allowed to identify two subgroups of patients with significant differences in survival (18,8 vs 7,5 months) [21].

Another relevant histological marker is the thyroid transcription factor-1 (TTF-1). The meta-analysis by Berghmans et al. showed that TTF-1 positivity was associated with a statistically significant longer survival in NSCLC: combined HR of eight studies was 0,64 and 0.53 in the adenocarcinoma subgroup [22]. We also found this impact of TTF-1 positivity both in univariate and multivariate analysis, with a MS of 15.4 months versus 6.45 months for negative TTF-1 patients (HR = 0.54), and believe that TTF-1 status is a reliable marker for survival.

Hypercalcemia is also a well-known adverse pronostic factor confirmed by our univariate analysis: median survival is 11.2 months vs 5 months for hypercalcemic patients (HR = 2.38; CI95% 0.96-5.88, p ≤ 0.008). In advanced stage, reported rates of hypercalcemia are of 12,5-35% [23], and 22% in the present study.

Indeed, if none of our patients experimented inaugural malignant hypercalcemia, preventing hypercalcemia during the disease course and incidence of second SRE is a major concern in this population.

Despite their inaugural revealing SRE, only 60% of our patients received biphosphonates. In previous phase III study, Rosen et al. demonstrated the long-term efficacy of zoledronic acid in preventing SRE in patients bearing skeletal metastasis [24]. Moreover, Hirsh et al. suggested that patients who had already experienced a SRE were at higher risk for subsequent SREs than patients with skeletal involvement without SRE. This over-risk was based on the SRE history of patients from Rosen’s phase III study evaluating zoledronic acid where this biphosphonate reduced the risk of SREs by 31%, prolonging the median time to first SRE by nearly 4 months [25]. In our study, 37% of the patients experienced a second SRE. Bae et al. reported a very closed rate of 33% second SRE in the population of 86 patients with first inaugural SRE [6] and a prescription rate of biphosphonates of 4.5%.

Administration of biphosphonates is here associated with an increased survival in univariate analysis- but not in multivariate analysis- with a median survival of 15.4 versus 5.2 months (HR = 0.4, CI95% 0.2-0.79; p = 0.001). Previous studies already showed a survival advantage of nearly 6 months for patients who had been treated with biphosphonates [26,27].

More recently, Denosumab, a human monoclonal antibody against RANKL, confirmed its superiority over zoledronid acid giving an overall survival improvement in a population of 811 NSCLC patients with bone metastases [28,29]. In conclusion, biphosphonates or denosumab should be early administrated in the course of the disease for these patients who were shown to be at higher risk of developing subsequent skeletal-related-event.

Finally, although the prognosis in NSCLC patients with inaugural SRE remains poor, respectively seven and two of the 55 patients ( 12,7% and 3.6%) are alive at 2 and 5 years. Case reports of long NSCLC survivors at 5 years with sole bone metastasis were reported with bone locations on distal extremities [30]. Bae et al. reported that patients with single bone metastasis had a survival 2.4 times longer than others [6].

A study by Okamoto [31] analysing predictive factor of long survival in stage IV non small cell lung cancer, found that multimodal treatment including a locoregional treatment like surgery, and early nodal status were significantly associated with longer survival. A long term improvement survival at 5 years can be achieved for patients after surgical resection of solitary metastases in brain or adrenal gland of non-small cell lung cancer [32]. We believe that an accurate early staging by PET-TDM and full body RMN may allow to better select oligometastatic patients in order to shift them to more aggressive therapy. Selected patients with single bone metastasis could benefit from a combination of surgical lung resection on the primitive tumor and intensified treatment on the skeletal metastasis, such as surgery and/or hypofractionated stereotactic body radiation therapy with dose escalation in a curative intent [33,34].

### Limitations

The major limitation of our study included the lack of recording of EGFR status, at this period of time (2003–2009). Our population may be heterogeneous regarding EGFR status, a known-predictive factor for OS and PFS [5,6]. Only two patients received EGFR TKI as first-line treatment, nowadays prescribed in EGFR-mutated patients. However, in the subgroup of twelve patients who received EGFR TKI as second or third line, median survival was 18 months, 2.5 times superior than for other treatment subgroups. Finally, there was a wide range of chemotherapy regimens and a selection bias of chemotherapy use, since 92% of our patients were selected through a chemotherapy registry.

## Conclusion

Despite its shortscomings, this retrospective study represents one of the largest follow-up of patients with an inaugural SRE revealing non-small lung cancer. Few patients with single bone metastasis and no visceral dissemination were able to achieve survival up to 5 years. In multivariate analysis, variables significantly associated with shorter overall survival are locally advanced T stage, weight loss ≥ 10%, inaugural spinal epidural metastasis, elevated C-reactive protein and negative TTF-1 status. Thus, a better selection of oligometastatic NSCLC patients likely to require or benefit from surgical procedure on inaugural skeletal metastasis can be made.

## Abbreviations

EGFR: Epithelial growth factor inhibitor; HR: Hazard ratio; NSCLC: Non small cell lung cancer; OS: Overall survival; PFS: Progression free survival; RANK: Receptor activator of nuclear factor kappa-B ligand; SEM: Spinal epidural metastasis; SRE: Skeletal-related event; TKI: Tyrosine kinase inhibitor; TTF-1: Thyroid transcription factor.

## Competing interests

The authors declare that they have no competing interests.

## Authors’ contributions

MD has conceived and designed this research and drafted the manuscript. SR and FG have supervised this work for analysis, interpretation of the data and drafting the manuscript. BB has performed the statistical analysis. CD and JC have participated in collecting the data at Curie Institute and Cochin Hospital Respiratory Disease Department. All authors read and approved the final manuscript.

## Pre-publication history

The pre-publication history for this paper can be accessed here:

http://www.biomedcentral.com/1471-2407/14/416/prepub
